# Development of a thiostrepton-free system for stable production of PLD in *Streptomyces lividans* SBT5

**DOI:** 10.1186/s12934-022-01992-1

**Published:** 2022-12-19

**Authors:** Juntan Wang, Haihua Zhu, Huiyi Shang, Bishan Guo, Mengxue Zhang, Fayun Wang, Lipan Zhang, Jun Xu, Hui Wang

**Affiliations:** 1 Institute of Business Scientific, Henan Academy of Sciences, 87 Wenhua Road, Zhengzhou, 450002 Henan China; 2 College of Life Sciences, Henan Agricultural University, Zhengzhou, 450002 Henan China; 3 School of Public Health, Shanghai Jiao Tong University School of Medicine, Shanghai, 200025 China

**Keywords:** Phospholipase D, *Streptomyces*, Expression system, Thiostrepton, Plasmid stability

## Abstract

**Background:**

Phospholipase D (PLD) is highly valuable in the food and medicine industries, where it is used to convert low-cost phosphatidylcholine into high-value phospholipids (PLs). Despite being overexpressed in *Streptomyces*, PLD production requires expensive thiostrepton feeding during fermentation, limiting its industrialization. To address this issue, we propose a new thiostrepton-free system.

**Results:**

We developed a system using a combinatorial strategy containing the constitutive promoter kasOp* and PLD G215S mutation fused to a signal peptide *sigcin* of *Streptoverticillium cinnamoneum pld*. To find a candidate vector, we first expressed PLD using the integrative vector pSET152 and then built three autonomously replicating vectors by substituting *Streptomyces* replicons to increase PLD expression. According to our findings, replicon 3 with stability gene (*sta*) inserted had an ideal result. The retention rate of the plasmid pOJ260-*rep*3-pld* was 99% after five passages under non-resistance conditions. In addition, the strain SK-3 harboring plasmid pOJ260-*rep*3-pld* produced 62 U/mL (3.48 mg/g) of PLD, which further improved to 86.8 U/mL (7.51 mg/g) at 32 °C in the optimized medium, which is the highest activity achieved in the PLD secretory expression to date.

**Conclusions:**

This is the first time that a thiostrepton-free PLD production system has been reported in *Streptomyces.* The new system produced stable PLD secretion and lays the groundwork for the production of PLs from fermentation stock. Meanwhile, in the *Streptomyces* expression system, we present a highly promising solution for producing other complex proteins.

## Background

Phospholipids (PLs) are a class of substances with distinct physiological properties. These are necessary for cell survival and metabolic regulation. Furthermore, as emulsifiers, cosmetic components, pharmaceutical preparations, and liposome preparations, PLs have great commercial value [1, 2]. The use of enzymes in the synthesis of PLs has several advantages, including mild reaction conditions and environmental friendliness, making it a promising option for large-scale industrial production [3, 4]. Phospholipase D (PLD, E.C. 3.1.4.4) is a key enzyme in the biocatalytic synthesis or modification of PLs, allowing the transphosphatidylation of high levels of phosphatidylcholine (PC) into less naturally occurring PLs [5, 6]. Because PC is abundant in nature, the main challenge is producing highly active PLD enzymes.

PLD natively produced by *Streptomyces* is more suitable for efficient production due to its higher transphosphatidylation activity and broader substrate specificity than that obtained from plant and animal sources [7]. Especially the *Streptomyces antibioticus* PLD, which has the advantage of a high specific activity [8, 9]. However, the yield of natural PLD is extremely low to meet commercial demands. Therefore, the large-scale expression of PLD through cell factories is of great value.

In the last two decades, researchers have made many attempts to express recombinant PLD in different hosts (Table 1). *Escherichia coli* is the most common host to express PLD because of its clear genetic background, high expression capacity, and rapid growth. But in fact, it is unsuitable for industrial production due to poor protein solubility, difficulty in extraction, and cytotoxicity for the cells [10–13]. Despite attempts to express PLD in other hosts such as *Bacillus subtilis* [14–16], *Pichia pastoris* [17, 18], and *Corynebacterium glutamicum* [19], the results were unsatisfactory, and PLD activity was generally low. *Streptomyces lividans* is well-known for its ability to produce structurally complex proteins as well as its high enzyme secretion capacity [20, 21]. Ogino et al. overexpressed *Stv. cinnamoneum* PLD in *S. lividans* 1326 (enzyme activity 20 U/mL) [22], demonstrating the potential of *S. lividans* as a host for PLD production. Since then, the enzymatic activity of PLD has increased constantly. Overexpression of *Streptomyces racemochromogenes* PLD (enzyme activity 30 U/mL) and *Streptomyces halstedii* PLD (enzyme activity 69.12 U/mL) has been achieved in *S. lividans*, respectively [18, 23]. *S. lividans* SBT5 is an optimized strain with a clear secondary metabolic background and does not produce endogenous pigments or antibiotics, which can be a superior host for PLD expression [24]. However, the problem is that thiostrepton is widely used as an inducer or antibiotic in these systems, and its high price limits the large-scale industrial production of PLD.

**Table 1:** Heterologous expression of *Streptomyces* PLD in different hosts

Hosts	PLD source	Secretory expression	Activity after optimization (U/mL)	References
*E. coli* BL21	*S. antibioticus*	–	12	[10]
*E. coli* BL21	*S. halstedii*	–	1.21	[11]
*E. coli* BL21	*S. chromofuscus*	–	122.94	[18]
*P. pastoris*	*S. halstedii*	+	2.36	[18]
*P. pastoris*	*Streptomyces* sp. PMF	+	0.22	[19]
*B. subtilis*	*Streptomyces* sp. PMF	+	0.14	[19]
*C. glutamicum*	*Streptomyces* sp. PMF	+	1.9	[19]
*S. lividans*	*Stv. cinnamoneum*	+	50	[22]
*S. lividans* TK23	*S. racemochromogenes*	+	30	[23]
*S. lividans* TK24	*S. halstedii*	+	68.33	[18]

Thiostrepton was discovered as an antibiotic in the *Streptomyces* system; it was later discovered to function as an inducer, promoting the binding of the activator TipAL to its promoter *tipA*, and catalyzing specific transcription [25, 26]. Although thiostrepton can increase the expression of target proteins, the constant feeding of thiostrepton in fermentation production is costly and contrary to green production concepts. Tao et al. attempted to replace thiostrepton by inserting the constitutive promoter ermEp* downstream of the inducible promoter *tipA*; however, without the inducer, PLD activity was only 13.41 U/mL [18]. Therefore, there is an urgent need to develop a novel thiostrepton-free system to achieve efficient secretory expression of PLD.

Compared to inducible expression, constitutive expression is more suitable for industrial process production because of its simplicity and efficiency. Several constitutive promoters have been used in *Streptomyces*, the most commonly used is ermEp* [27]. The kasOp* is an engineered derivative of the *kasOp* regulating the *Streptomyces* antibiotic regulatory protein (SARP) gene in *S. coelicolor*, which exhibits higher levels of actinorhodin production than ermEp* and *SF14p* controlling *actII*-ORF4 expression. In addition, it has been used to overexpress ε-PL synthase and xylose/glucose isomerases [28–30]. However, the use of kasOp* for PLD expression has not yet been reported.

An appropriate vector will facilitate the successful expression of the target protein. Compared to *Streptomyces* initial plasmids such as SCP2, SCP2*, and SLP1.2, pIJ101 and its derivatives have a high copy number and broad host range [31, 32]. The pIJ101-derived vectors are widely used, including efficient shuttle vectors and vectors for gene deletion [33–35]. The genetic stability of plasmids is also necessary for the expression system. An approximately 1.2 kb fragment downstream of the pIJ101 replication region is known to be inextricable to the plasmid stability. The fragment included the stability gene (*sta*), a 0.5 kb sequence between the PstI site at position 31 and the SstII site at position 33 [31], and a strong incompatibility site (*sti*, 0.2 kb), which has an impact both on plasmids’ genetic stability and copy number [36, 37]. However, it was sparingly utilized in protein expression. In this study, we optimized the *S. antibioticus* PLD, selected a constitutive promoter kasOp* instead of the conventional inducible promoter *tipA,* and constructed new shuttle vectors with *sti* or *sta* sequences inserted to build a thiostrepton-free system, which finally achieved a stable efficient expression of PLD in *S. lividans* SBT5.

## Results

### Construction of a thiostrepton-free PLD expression system

**Fig. 1:**
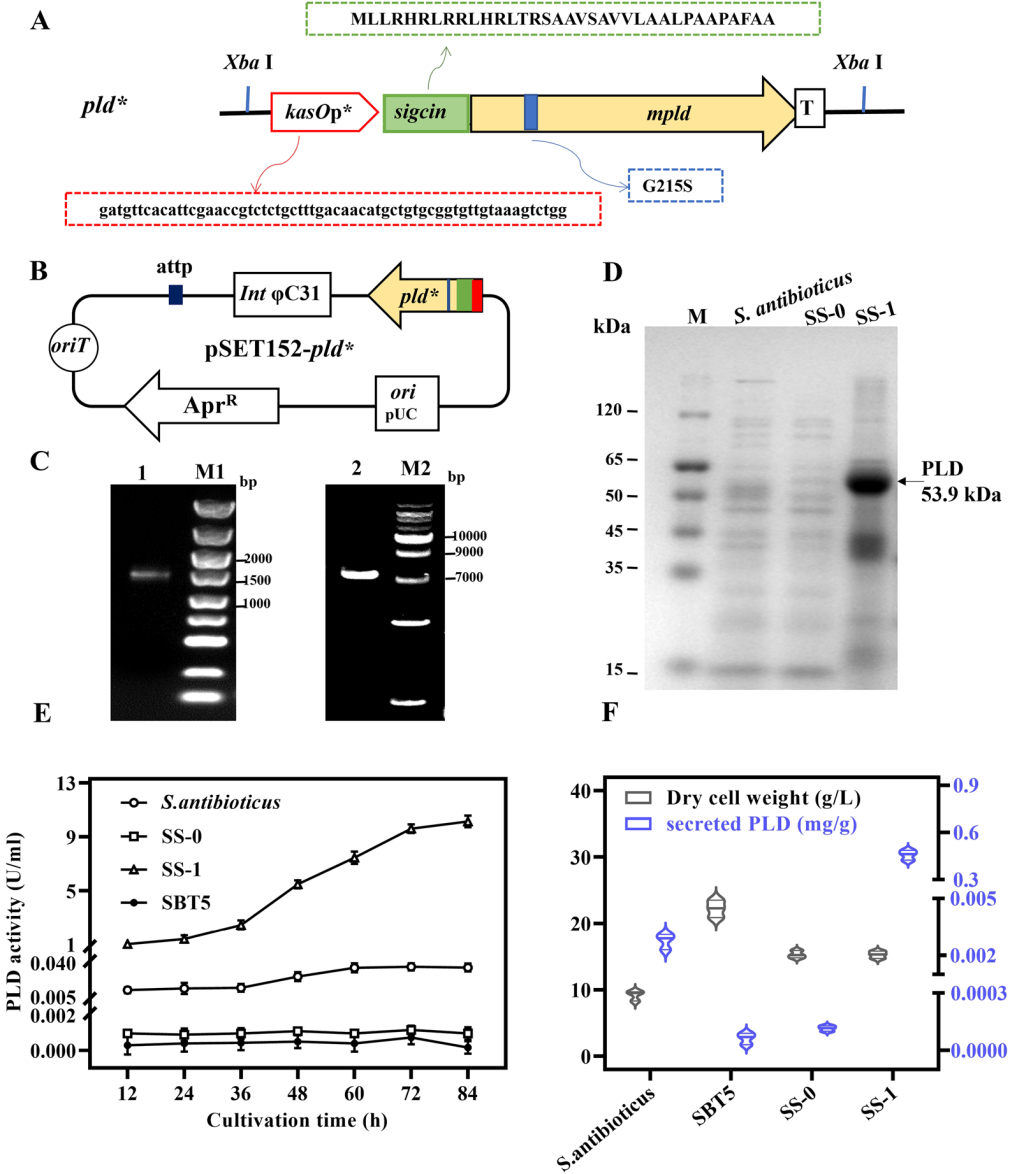
Construction of thiostrepton-free PLD secretory expression system. **A** Schematic diagram of the optimized gene pld*. kasOp*: constitutive promoter derivative of *kasOp*; *sigcin*: signal peptide derived from *Stv. cinnamoneum pld*; *mpld*: PLD gene codon optimized with G215S mutated. **B** Schematic diagram of the integrative PLD expression plasmid pSET152-pld*. **C** DNA gel electrophoresis analysis. lane 1: restriction fragment of pld*, 1.8 kb; M1: DL2000 DNA ladder; lane 2: restriction fragment of pSET152-pld*, 7.5 kb; M2: 1-kb DNA ladder. **D** SDS-PAGE analysis of culture supernatants after 84 h of fermentation with various strains. **E** Time-course of the PLD activity of different strains (SS-0: SBT5/pSET152, SS-1: SBT5/pSET152-pld*). All data are the average of three independent experiments with an indication of standard deviations. **F** Dry cell weight and PLD secretion efficiency of different strains

By modifying genetic elements, a thiostrepton-free system for PLD expression was created. To improve PLD's transphosphatidylation activity, the gene encoding *S. antibioticus* PLD was codon optimized and mutated by introducing a G215S substitution [38]. PLD's native signal peptide was replaced with *sigcin*, a more efficient secretion signal peptide derived from *Stv. cinnamoneum pld* [39, 40]. The optimized sequence kasOp*-*sigcin*-*pld* (pld*) (Fig. 1A) was subcloned into the integrative vector pSET152 [41, 42], which is targeted for integration in the *Streptomyces* chromosome via the *att/int* system of phage φC31 at the XbaI site (Fig. 1B, C), and the PLD expression plasmid pSET152-pld* was conjugated to the host *S. lividans* SBT5 to obtain the recombinant strain SBT5/pSET152-pld* (SS-1). SBT5/pSET152 (SS-0) was used as a control strain. In both, apramycin was used for the selection.

Significant changes were observed in the total protein profile. The molecular mass was 54 kDa, as shown by SDS-PAGE (Fig. 1D, SS-1), which coincides with the deduced PLD without signal peptide (53.9 kDa). The wild-type *S. antibioticus* and control strain SS-0 showed little or no secretion of PLD, while significant levels of secreted PLD were detectable in strain SS-1 (Fig. 1D). The extracellular PLD activity of wild-type *S. antibioticus* was approximately 0.036 U/mL, which was generally in agreement with the report in the literature of 0.034 U/mL [8]. The extracellular PLD activity of strain SS-1 was approximately 9.85 U/mL, which was 273 times greater than the wild-type strain, while the productivity of the control strain SS-0 was largely undetectable (Fig. 1E). The biomass accumulation and PLD secretion efficiency of different strains were compared using MM medium fermented for 84 h under the same conditions. The wild strain *S. antibioticus* had the lowest cell dry weight, and the recombinant strains SS-1 and SS-0 had similar dry cell weights (~ 15.3 g/L), lower than the host SBT5 and at an intermediate level. Unlike biomass accumulation, PLD secretion efficiency was intermediate in *S. antibioticus* and lower in strain SS-0 and host SBT5. The strain SS-1 had the highest PLD secretion efficiency of 0.46 mg/g (Fig. 1F).

### Construction of autonomously replicating vectors and PLD expression

**Fig. 2:**
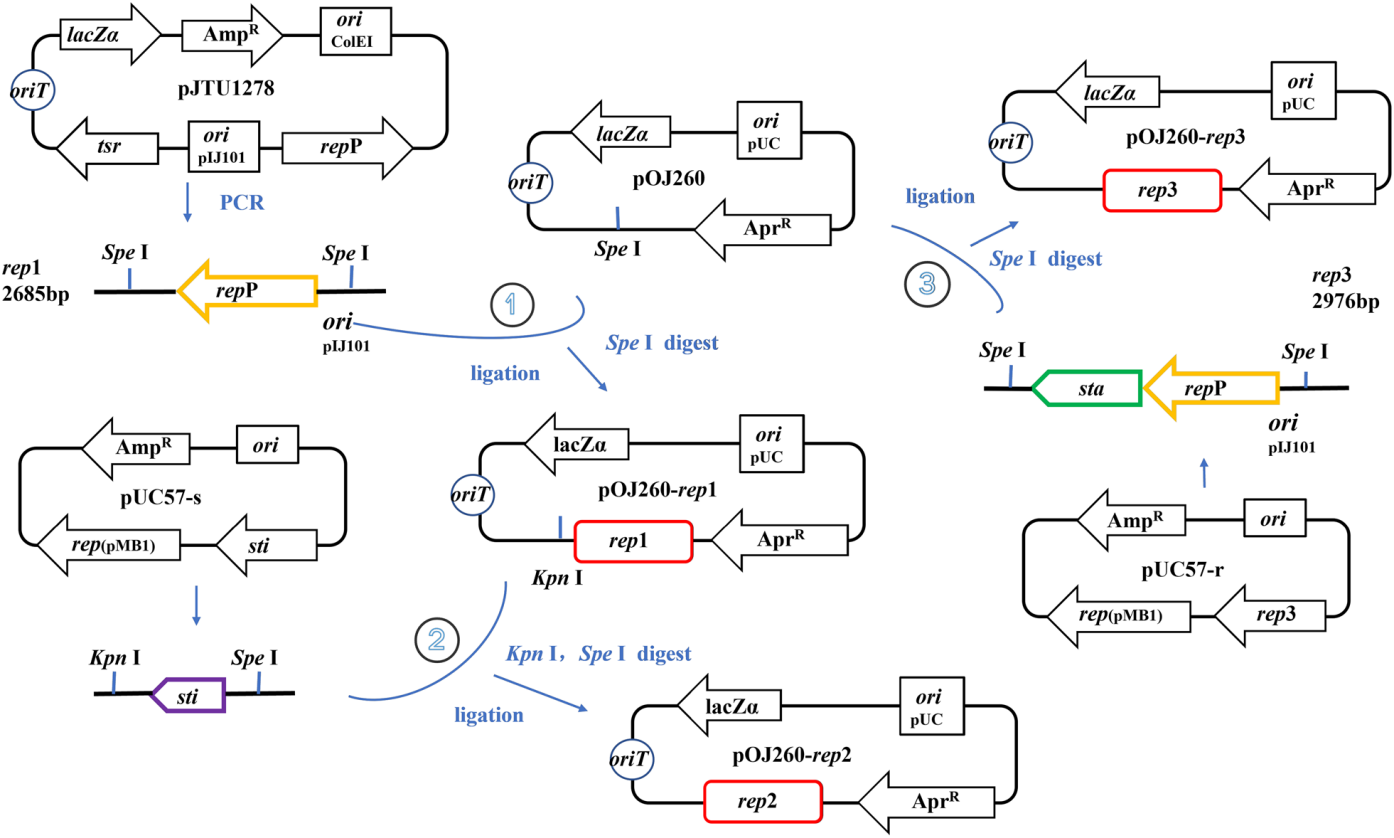
Schematic diagram of pOJ260-*rep*n construction. The replicon *rep*1 of pIJ101 was obtained by PCR amplification using plasmid pJTU1278 as the template, SpeI subcloned site into the plasmid pOJ260 to obtain autonomously replicating vector pOJ260-*rep*1; Fragment *sti* was digested from plasmid pUC57-s at KpnI and SpeI, the same site was ligated to vector pOJ260-*rep*1 to obtain the vector pOJ260-*rep*2; replicon *rep*3 contains *sta* sequence was digested from plasmid pUC57-r at SpeI site, the same site was ligated to the plasmid pOJ260 to obtain the vector pOJ260-*rep*3

**Fig. 3:**
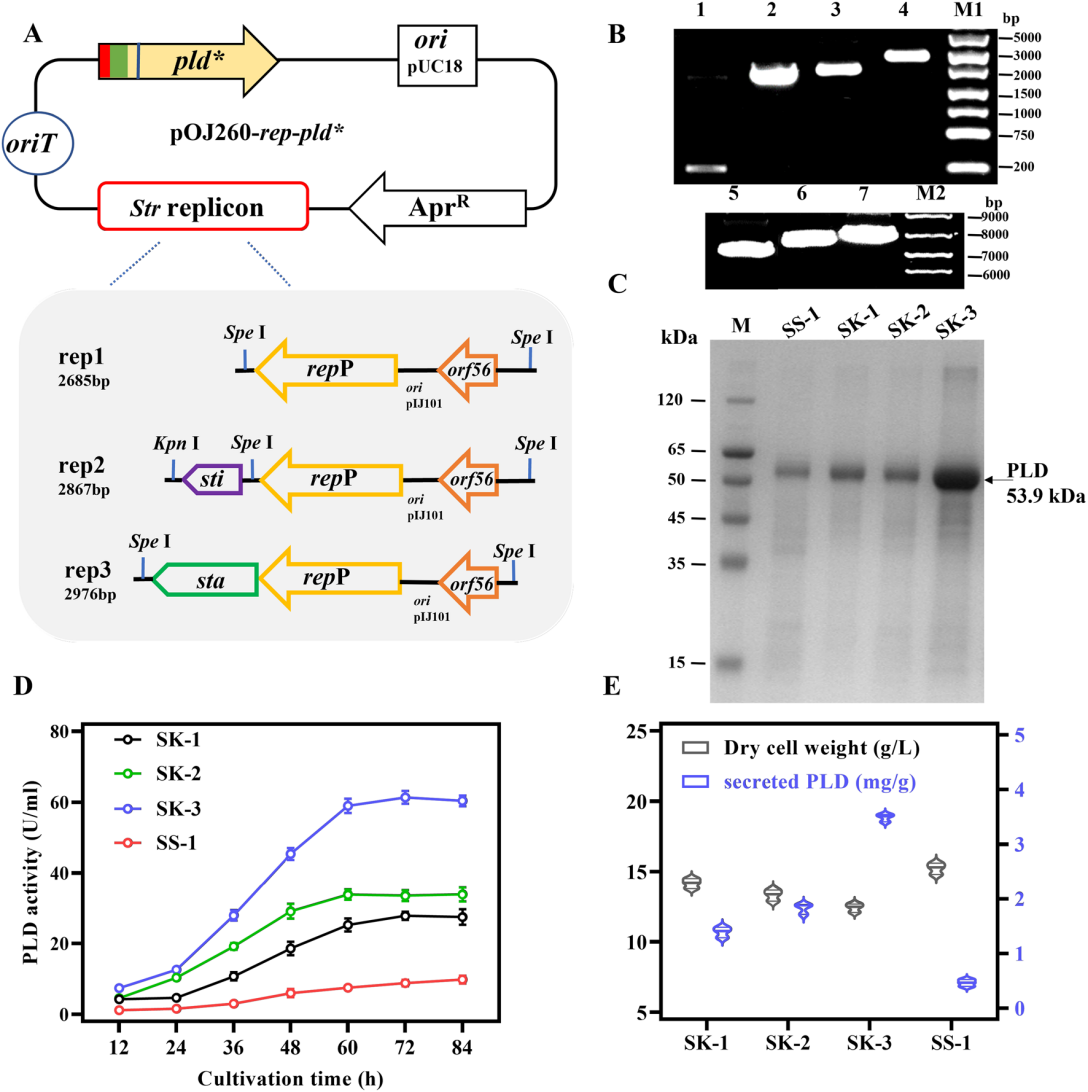
Construction of the PLD secretory expression system. **A** Schematic diagram of plasmid pOJ260-*rep*n-pld*. The replication protein gene *rep*P and the replication initiation region *ori* from the original *Streptomyces* plasmid pIJ101 are present in all three replicons. Additionally, *rep*2 contains a 0.2 kb *sti* fragment, and *rep*3 has an approximately 0.62 kb sequence of *sta*. **B** DNA gel electrophoresis analysis. lane 1: restriction fragments of *sti*; lane 2: restriction fragments of *rep*1; lane 3: restriction fragments of *rep*2; lane 4: restriction fragments of *rep*3; M1: DL5000 DNA ladder; lane 5, lane 6, and lane 7 represent linearized plasmids of pOJ260-*rep*1-pld*, pOJ260-*rep*2-pld*, pOJ260-*rep*3-pld*, with sizes of 7.9 kb, 8.1 kb, 8.2 kb, respectively; M2: 1 kb DNA ladder. **C** SDS-PAGE analysis of culture supernatants after fermentation of 84 h with different strains (SK-1: SBT5/pOJ260-*rep*1-pld*, SK-2: SBT5/pOJ260-*rep*2-pld*, SK-3: SBT5/pOJ260-*rep*3-pld*). **D** PLD activity was measured over time in various strains. All data are the average of three independent experiments, with standard deviations indicated.** E** Dry cell weight and PLD secretion efficiency of different strains

Although the recombinant strain SS-1 carrying the integrative plasmid pSET152-pld* was thiostrepton-free, the activity of PLD was still low from the perspective of industrial production. To improve the PLD secretion efficiency, different pIJ101-based *Streptomyces* replicons were added to the plasmid pOJ260 [42] to create the new autonomously replicating shuttle vector pOJ260-*rep*n (Fig. 2). The expression plasmid pOJ260-*rep*n-pld* was obtained by inserting the pld* fragment into the vector pOJ260-*rep*n (Fig. 3A, B). The new expression plasmids were *Apr*-resistant and could be selected in both *E. coli* and *Streptomyces*. Recombinant strain SBT5/pOJ260-*rep*n-pld* (SK-n) was obtained by *E. coli-Streptomyces* conjugational transfer into the host *S. lividans* SBT5.

The SDS-PAGE analysis by ImageJ showed that the expression of PLD was abnormally high, accounting for more than 95% of the soluble protein in the supernatant (Fig. 3C). The strain SK-3 had the highest PLD secretion efficiency of all. The PLD activity increased rapidly after 24 h and then plateaued after 60 h. Strains SK-1, SK-2, and SK-3 secreted about 28 U/mL (1.39 mg/g), 34 U/mL (1.82 mg/g), and 62 U/mL (3.49 mg/g) PLD, respectively, and were significantly higher than that of strain SS-1 (9.85 U/mL, 0.46 mg/g). However, the biomass accumulation of strains SK-1, SK-2, and SK-3 was approximately 14.2, 13.3, and 12.6 g/L, respectively, and was lower than that of the strain SS-1 (15.3 g/L). The results showed that the biomass of the strains was inversely related to the efficiency of PLD secretion (Fig. 3D, E).

### Genetic stability of new shuttle vectors

**Fig. 4:**
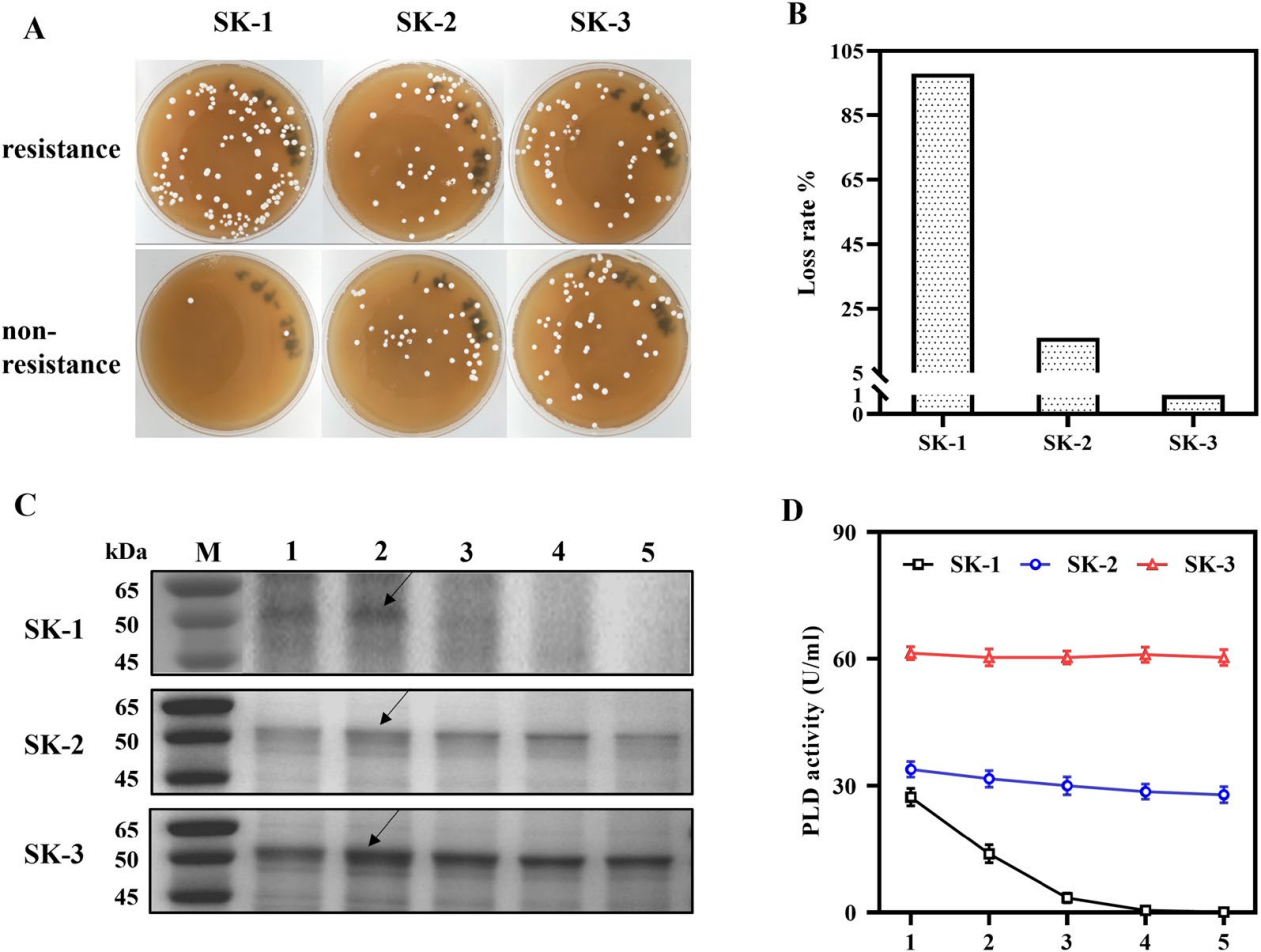
Determining the stability of autonomously replicating plasmids. **A** Growth of spores of the fifth passage on MS plates with and without antibiotics. **B** Plasmid loss rate after five passages with no antibiotics added. **C** SDS-PAGE analysis of PLD in various passages without the addition of antibiotics. Lane M: protein marker; lane 1–5: passage numbers of the strains. **D** Changes in the PLD activity of recombinant strains in different passages without the addition of antibiotics. All data are the average of three independent experiments, with standard deviations indicated

Because PLDs are widely used in food and pharmaceuticals, the restriction of antibiotics is increasing in industrial production. However, many described expression vectors must contain antibiotic-resistance genes as selection markers. To improve the expression system even further, *Streptomyces* replicon variants from pIJ101 were tested for genetic stability by resistance loss. Autonomously replicating vectors have a higher copy number than integrative vectors generally, but may lose during passaging. To enhance the stability of plasmids, a *sti* fragment was added to *rep*2 [36]. In addition, *rep*3 is derived from plasmid pIJ6021 and contains an approximately 0.62 kb *sta* sequence [31, 33]. The genetic stability of the plasmid pOJ260-*rep*1-pld* was unsatisfactory in the absence of apramycin, with 98% plasmid loss after five culture passages; pOJ260-*rep*2-pld* with a 16% loss rate and pOJ260-*rep*3-pld* with almost no loss (Fig.4A, B). The change in PLD expression in the five no-resistance passages revealed that the strain SK-1 secreted 2.76 U/mL PLD activity in the third passage, which decreased by 90%. In the fifth passage, SK-2 lost about 18% of its PLD activity of 27.86 U/mL, which was roughly the same as the loss rate (Fig. 4C, D).

### Optimization of carbon and nitrogen sources

**Fig. 5:**
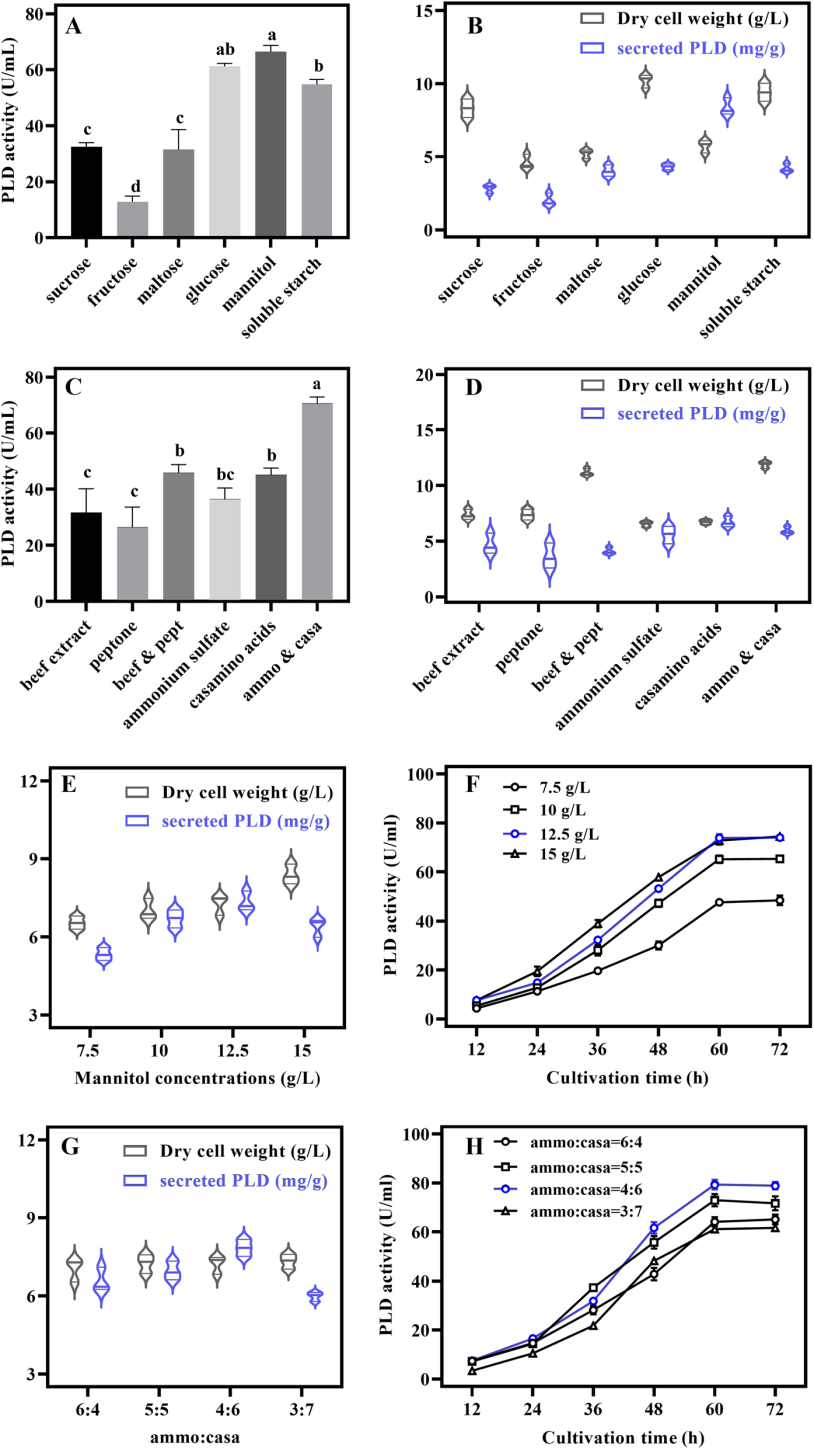
Optimization of carbon and nitrogen sources. **A** Effect of different carbon sources on PLD activity. Each concentration was 10 g/L. Columns with the same letters are not significantly different. Different lowercase letters above columns indicate statistical differences at p < 0.05. **B** PLD secretion efficiency and dry cell weight under different carbon sources.** C** The effect of various nitrogen sources on PLD activity. Each concentration was 10 g/L, and the complex nitrogen source concentration was 1:1. **D** Under different nitrogen sources, dry cell weight and PLD secretion efficiency were measured.** E** Dry cell weight and PLD secretion efficiency at various mannitol concentrations. **F** Optimization of the mannitol concentration in PLD activity. **G** Dry cell weight and PLD secretion efficiency under different ratios between ammonium sulfate and casamino acids. **H** Optimization of the ratio between ammonium sulfate and casamino acids in PLD activity. All data are the average of three independent experiments with standard deviations indicated

The fermentation conditions of the recombinant strain SK-3 were optimized to further increase the PLD production. Different microbes obtain nutrients from various sources. The excessive secretion is stimulated when carbon backbones cannot be metabolically funneled toward cell growth [43, 44]. We sought a nutrient source that would result in higher PLD activity and lower bacterial mass in order to reduce industrial waste disposal while also improving production efficiency. The best carbon source was mannitol, which resulted in 66 U/mL PLD activity after 72 h. The PLD activity was increased by about 8%, and the bacterial mass was decreased by 44%. At the same time, the efficiency of PLD secretion is maximized (Fig. 5A, B). The results of the optimization of the nitrogen source revealed that the combination nitrogen was better than the single nitrogen, and the most effective nitrogen source was ammonium sulfate with casamino acids, resulting in a PLD activity of 72.9 U/mL, and the bacterial mass was relatively low. Due to their low biomass, casamino acids was a better nitrogen source than complex nitrogen sources in terms of PLD secretion efficiency (Fig. 5C, D). However, in terms of PLD production, the composite nitrogen source ammonium sulfate with casamino acids was eventually selected.

Further fine-tuning of the mannitol concentration showed that a similar PLD activity was obtained, i.e., 75 U/mL, at 12.5 g/L and 15 g/L, which increased the PLD activity by 19%. The biomass gradually increased with increasing mannitol concentration, and the secretion efficiency of PLD was highest when 12.5 g/L mannitol was added. However, when the mannitol concentration was increased to 15 g/L, the efficiency of PLD secretion decreased (Fig. 5E, F). As a result, 12.5 g/L was used as the mannitol concentration in subsequent experiments. Further optimization of the ratio of ammonium sulfate with casamino acids resulted in a maximum PLD activity of 80.6 U/mL, an increase of about 10%, when ammonium sulfate and casamino acids were 4 g/L and 6 g/L, respectively. The adjustment of the ratio did not have a significant effect on biomass. However, increasing the amount of casamino acids, the PLD activity increases, reaching a maximum at 4:6. When the ratio of casamino acids continued to increase, enzyme activity and PLD secretion efficiency would decline significantly (Fig. 5G. H).

### Optimization of temperature

**Fig. 6:**
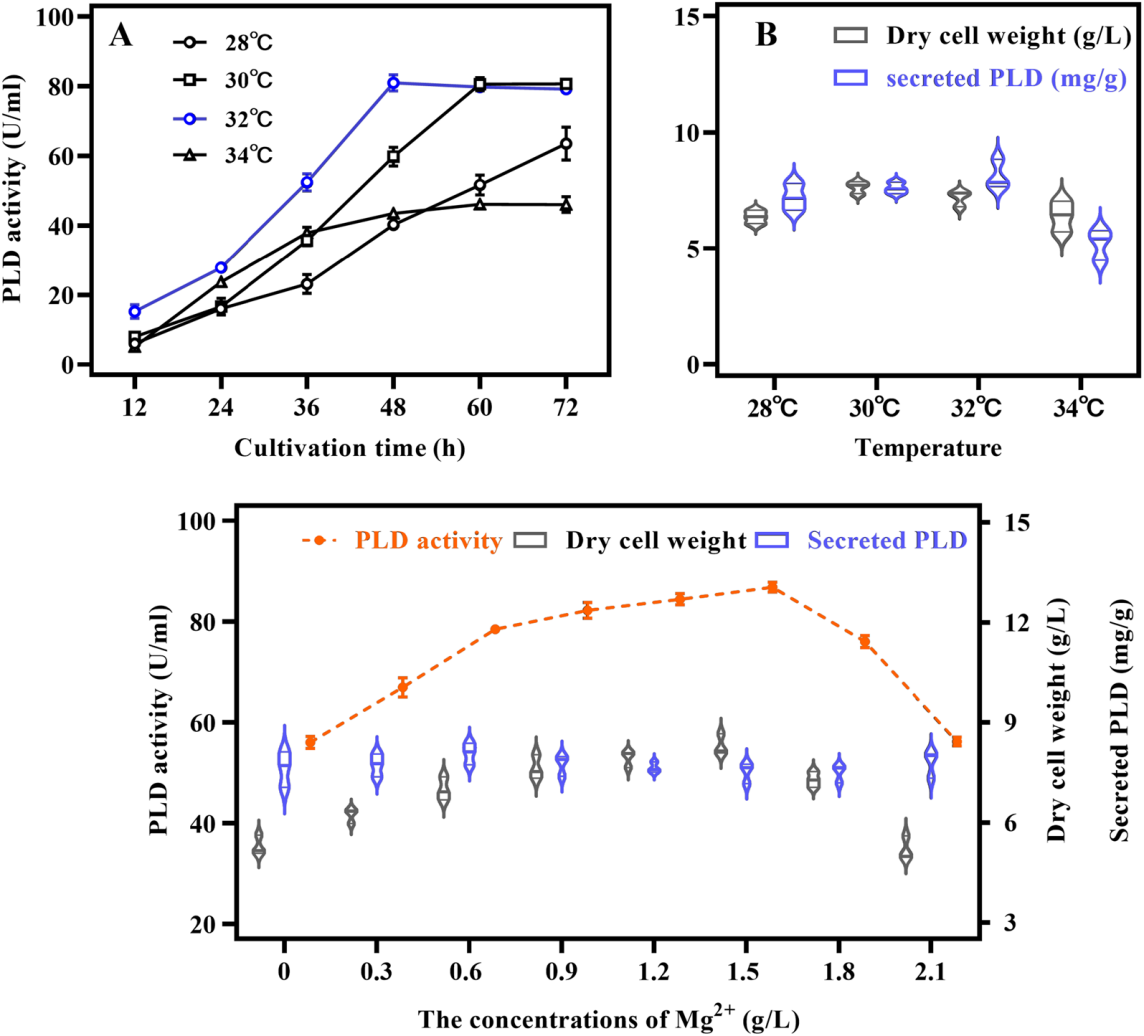
Optimization of fermentation conditions. **A** Optimization of incubation temperature in PLD activity. **B** Dry cell weight and PLD secretion efficiency at various temperatures. **C** Optimization of Mg^2+^ concentration in PLD activity, dry cell weight, and PLD secretion efficiency. All data are the average of three independent experiments, with standard deviations indicated

The time course of PLD production was studied using fermentation of strain SK-3 at different temperatures based on the optimal carbon and nitrogen sources. At 34 °C, the PLD activity was 48 U/mL, showing a decrease of about 44% compared to that at 30 °C, indicating that high temperature was not conducive to enzyme production. At 28 °C, the PLD activity did not reach the plateau at 72 h, increasing the incubation time. At 32 °C, the PLD activity was comparable to that at 30 °C, approximately 80.9 U/mL; however, the plateau time decreased by 12 h compared to the 30 °C conditions to 48 h after fermentation. In addition, it was more suitable for protein expression at 32 °C, which may be burdensome to cell growth and affect biomass (Fig. 6A, B). As a result, 32 °C was chosen as the incubation temperature to reduce fermentation time and thus improve industrial production efficiency.

### Optimization of Mg^2+^ concentration

Based on the above optimization, an appropriate amount of Mg^2+^ contributes to proper cell growth and protection. The effect of adding different concentrations of Mg^2+^ to the culture medium on the expression of PLD was investigated. The results showed that the PLD activity and the bacterial mass increased with the increase in Mg^2+^ concentration, and the change in PLD secretion efficiency was not significant. The bacterial mass was 8.3 g/L, and the PLD activity was 86.8 U/mL at 1.5 g/L Mg^2+^. The bacterial mass increased by about 19% compared to the pre-optimization (6.99 g/L, 78.4 U/mL), and the PLD activity increased by 11%. In comparison to the absence of Mg^2+^ addition (5.29 g/L, 56 U/mL), the bacterial mass increased by 56%, and PLD activity increased by about 55% (Fig. 6C).

## Discussion

Researchers expressed recombinant PLD in different hosts (Table 1). Although Wu et al. could successfully express soluble PLD in *E. coli* by lactose feeding and low-temperature induction [11], the intracellular expression was not conducive to the industrial extraction of PLD. In hosts such as *P. pastoris*, *B. subtilis,* and *C. glutamicum*, although the secretory expression of the protein was achieved, the activity of PLD was considerably lower than that of the *Streptomyces* hosts, probably because PLD hydrolyzed phospholipids on the cell membrane and disrupted normal cell membrane function [16, 17, 19]. *Streptomyces* is a natural host for PLD; PLD can be folded properly and modified efficiently after translation in *Streptomyces* without forming inclusion bodies and with little effect on cell growth. It can be seen that *Streptomyces* hosts have a high potential for industrial PLD production.

Initially, researchers expressed PLD in *S. lividans* using the original promoter of PLD [22, 23] and later introduced efficient promoters such as *tipA*, *SF14p*, *hrdBp,* and ermEp* from *Streptomyces* [18, 45, 46]. According to our findings, the strong promoter kasOp* significantly increased PLD expression. PLD activity and secretion efficiency of recombinant strain SS-1 were increased approximately 273-fold and 165-fold, respectively, when compared to the wild strain. In addition to promoters, the choice of vector plays an important role in PLD expression. The *Streptomyces* replication region of the autonomously replicating vector pOJ260-*rep*n is derived from the original *Streptomyces* plasmid pIJ101 which has a higher copy number than the integrative vector pSET152. When pSET152 was used to express pld*, the PLD activity of strain SS-1 was approximately 9.85 U/mL, whereas the PLD activities of strains SK-1, SK-2, and SK-3 were 2.84, 3.45, and 6.29 times that of SS-1, respectively. The final PLD activity was 3.1 times greater than that reported by Ogino et al. (20 U/mL, unoptimized fermentation conditions), which is adequate for industrial production.

The stability of plasmids in host cells is one of the most vital factors in protein industrial production achievement. There is little research in *Streptomyces* expression systems on the genetic stability of plasmids with non-antibiotic selection. In 2013, Sevillano et al. developed a toxin-antitoxin system for proteins’ stable expression [47]. Unlike the *yefM/yoeBsl* system of *S. lividans*, we improved the stability of the expression plasmids by adding *sti* or *sta* sequences to the replicon. During PLD expression, strain SK-1 showed a significant loss of plasmid without added antibiotics, whereas SK-2 and SK-3, containing the *sti/sta* sequence, were relatively genetically stable. Compared to the *sti* sequence, the *sta* sequence significantly affected the plasmid’s genetic stability. In addition, PLD secretion of the strain SK-3 was the highest among all recombinant strains, but the reason is unclear for its efficient expression. Since the negative regulator *cop* controls plasmid copy number in trans and only acts on the natural orientation of *sti*^+^ plasmid [36], it could explain why strain SK-2 secreted more PLD than SK-1. However, the effect of the *sti* sequence on PLD efficient expression does not seem as significant as we expected.

After analyzing the biomass and secretion efficiency of different strains, we found that the low expression group of PLD (SS-1, SS-1) did not affect cell growth, which is consistent with Hamed et al. [48]. But in the high expression group (SK-1, SK-2, SK-3), cell growth was inhibited with the increase in PLD expression level. Differences in biomass could be due to the high expression of PLD [49]. However, this is uncertain to high expression of PLD did not seem affected cell growth in the results of Tao et al. [16]. In addition, changes in protein abundance may also be one of the reasons why cell growth is affected.

The fermentation cycle was shortened, and production efficiency was improved when the culture temperature was raised sufficiently. Intracellular overexpression of PLD in *Brevibacillus* causes a highly enhanced Ca^2+^ influx, disturbing the ionic homeostasis of the cell membrane and causing cell damage or death [50]. The addition of appropriate Mg^2+^ to the medium might exert a protective effect on the cells, resulting in a final increase in PLD activity for the recombinant strain SK-3. This study’s conditions for strain SK-3 were based on a 250 mL shake flask fermentation, and the fermenters used for production are generally better than shake flasks; thus, there is a potential to further enhance PLD activity.

## Conclusion

In this study, a new thiostrepton-free system was built to achieve an efficient and stable expression of PLD using *S. lividans* SBT5 as the host. We optimized the secretion of PLD by adapting genetic elements, including but not limited to the kasOp* promoter, the *sigcin* signal peptide, the codon optimization, and the PLD G215S mutation. We constructed a new autonomously replicating vector pOJ260-*rep*3, which was proven stable and efficient. After the fermentation optimization, the PLD enzyme activity of the recombinant strain SK-3 finally reached 86.8 U/mL, approximately a 40% improvement from pre-optimization (62 U/mL), which was the highest of the secreted type reported. We believe the study will lay the groundwork for further fermentation system development and facilitate the industrial production of PLDs. We will improve the repeated utilization of PLD in the future by immobilizing enzymes and actively promoting its use in the production of rare phospholipids. Furthermore, we will improve the system by expressing other proteins in order to set up a super-efficient *Streptomyces* expression system.

## Materials and methods

### Strains, plasmids, and culture conditions

Table 2 lists the strains and plasmids used in this study. *S. lividans* SBT5 was obtained by knocking out the *act, red, cda* biosynthetic gene clusters (BGCs) from *S. lividans* TK24 and integrating the global regulatory gene *afsRS*_cla_ into the site of the *cda* gene cluster [24]. Plasmids pUC57-p, pUC57-s, and pUC57-r were synthesized by Jinsrui Biotechnology Co., Ltd (Nanjing, China). *E. coli* strains were cultured at 37 °C (200 rpm) in the Luria–Bertani (LB) medium. *Streptomyces* strains were cultured at 30 °C for 7 days on mannitol soy flour (MS) agar plates (20 g of soy flour, 20 g of mannitol, and 20 g agar per L) for conjugation and sporulation. For fermentations, 1% spore suspension was inoculated into a test tube containing 5 mL MM medium (10 g glucose, 5 g of (NH_4_)_2_SO_4_, 5 g of casamino acids, 3.4 g of K_2_HPO_4_·3H_2_O, 2.34 g of NaH_2_PO_4_·2H_2_O, 0.6 g MgSO_4_·7H_2_O per L, trace metal ions ZnSO_4_·7H_2_O, FeSO_4_·7H_2_O, MnCl_2_·4H_2_O and CaCl_2_ were all 0.001 g/L) and grown at 30 °C (220 rpm) for 2 days. If not otherwise specified, a 1 mL aliquot of the culture was inoculated into a 250 mL flask containing 100 mL of the MM medium and grown at 30 °C (220 rpm) for 3 days. Ampicillin (70 µg/mL), thiostrepton (50 µg/mL), apramycin (50 µg/mL), and trimethoprim (30 µg/mL) were the antibiotics used. DNA and transformation techniques for *E. coli* and *Streptomyces* were performed according to the methods described by Green and Sambrook. [51] and Kieser et al. [52], respectively.

**Table 2:** Strains, genes, and plasmids used in this study

Strains/genes/plasmids	Characteristics	Reference
Strains		
* E. coli* DH5α	General cloning host	[53]
* E. coli* ET12567/pUZ8002	Methylation-deficient strain, for *E. coli-Streptomyces* interspecies conjugation	[54]
* S. antibioticus*	Wild-type strain	[9]
* S. lividans* SBT5	Expression host. *S. lividans* TK24 △*act*△*redKL*△*cdaPS3-SLI3600*::*afsRS*_*cla*_	[24]
Genes		
kasOp*	Constitutive strong promoter for gene overexpression in *Streptomyces* (GenBank: MN306530)	[30]
* sigcin*	*Stv. cinnamoneum pld* (GenBank: BAA75216) derived signal peptide	[39]
* mpld*	*S. antibioticus pld* (GenBank: BAA03913) was codon-optimized, G215S mutation	[38]
pld*	kasOp*-*sigcin*-*mpld*	
* sti*	*Streptomyces* strong incompatibility region (GenBank: AY667410)	[36]
* sta*	The sequence from pIJ101 between the PstI site at position 31 and the SstII site at position 33	[31]
* rep*3	Derived from the *Streptomyces* vector pIJ6021 (GenBank: AJ414669). *sta*, *rep*P, *orf56*	[33]
Plasmids		
pIJ101	Multi-copy broad host-range *Streptomyces plasmid* (GenBank: M21778) from *S. lividan*s ISP 5434	[31]
pSET152	φC31 based streptomyces integrative vector, Apr ^r^	[42]
pJTU1278	*Streptomyces* autonomously replicating vector, oriT (RK2), pIJ101 *ori*, ColE1 *ori*, Amp^r^, Tsr ^r^	[35]
pOJ260	Vector for gene disruption and transplacement, oriT (RK4), pUC18 *ori*, Apr^r^	[42]
pUC57	*E. coli* cloning vector	
pUC57-p	Artificially synthesized sequence pld* EcoRV cloned into vector pUC57, Amp^r^	This work
pUC57-s	Artificially synthesized sequence *sti * EcoRV cloned into vector pUC57, Amp^r^	This work
pUC57-r	Artificially synthesized sequence *rep*3 EcoRV cloned into vector pUC57, Amp^r^	This work
pOJ260-*rep*1	Fragment *rep* from pJTU1278 SpeI cloned into vector pOJ260. *rep*P, *orf56*, Apr^r^	This work
pOJ260-*rep*2	Fragment *sti* from pUC57-s KpnI/SpeI cloned into vector pOJ260-*rep*1. *sti*, *rep*P, *orf56*, Apr^r^	This work
pOJ260-*rep*3	Fragment *rep*3 from pUC57-r SpeI cloned into vector pOJ260. *sta*, *rep*P, *orf56*, Apr^r^	This work
pSET152-pld*	Fragment pld* from pUC57-p XbaI cloned into vector pSET152, Apr^r^	This work
pOJ260-*rep*n-pld*	Fragment pld* XbaI cloned into vector pOJ260-*repn*, Apr^r^. *rep*n: *rep*1*, rep*2*, rep*3	This work

### Plasmids construction

The new shuttle vector pOJ260-*rep*n was constructed by adding different *Streptomyces* replicons to the pOJ260 (Addgene, USA) backbone (Fig. 2A). The replicon *rep*1 (2.6 kb) was amplified by a ProFlex PCR System (Thermo Fisher Scientific, USA) using the following primer pair: 5′-TTGACTAGTCCAGGATTACTCCCGCGGCTTC-3′ and 5′-TTGACTAGTGATGGCGGATGGCTGCCCTGAC-3′ using the pJTU1278 (Addgene, USA) as the template, and inserted into the SpeI site of pOJ260 (pOJ260-*rep*1). The fragment *sti* (0.2 kb) from pUC57-s was inserted into the KpnI/SpeI sites of pOJ260-*rep*1 (pOJ260-*rep*2). The replicon *rep*3 (2.9 kb) from pUC57-r was inserted into the SpeI site of pOJ260 (pOJ260-*rep*3). The fragment pld* (1.8 kb) from pUC57-p was inserted into the XbaI site of pSET152 (pSET152-pld*) and pOJ260-*rep*n (pOJ260-*rep*n-pld*). DNA polymerase, restriction endonucleases, T4 DNA ligase, and DNA purification kits were purchased from Takara (Dalian, China).

### Genetic stability of recombinant strains

The first-passage strains' spore suspensions were incubated in MS for 6 days at 30 °C without antibiotics. The fifth-passage strains’ spore suspension was diluted 1000 times and incubated in anti-free MS plates for 6 days at 30 °C, after which grown colonies were counted. The number of colonies without antibiotics was counted as a, and those with antibiotics as b. The percentage of plasmid loss after five passages was calculated using a as the denominator and a-b as the numerator. To evaluate the stability of the recombinant strains in producing PLD without antibiotics, spore suspensions of each passage were inoculated in the MM medium and fermented.

### Protein assay

Supernatants of various transformants were harvested at different fermentation times from the fermentation broth by spinning the cells at 12,000 rpm for 10 min. Protein profiles were analyzed using 12% denaturing SDS-PAGE in a MiniProtean II system (Bio-Rad, USA). ImageJ software was used to assess the intensity of protein bands. The protein expression of PLD was calculated from the measured specific activity (1392 U/mg) and PLD activity.

### PLD assay

The PLD activity was assayed using PC as a substrate and measuring the formation of choline with choline oxidase and peroxidase by a spectrophotometric assay with minor modification [55]. A Full wavelength microplate reader was used to measure the absorbance (Tecan, Swiss). In a nutshell, 0.1 g of PC (Sigma, USA) was mixed with 10 mL of anhydrous ethanol and distilled water (1:1) and shaken in an ice bath until an emulsion was formed. Next, 200 µL was taken in a 5 mL centrifuge tube, and 200 µl assay solution (0.04 mol/L Tris–HCl pH 5.5, 0.01 mol/L CaCl_2_, and 1 g/L TritonX-100) was added. This mixture was preheated for 5 min at 37 °C Following that, 100 µL of the enzyme solution to be tested was immediately added and shaken (200 r/min) at 37 °C for 10 min. After that, 200 µL of the reaction termination solution (0.01 mol/L EDTA, 10 g/L 16-alkyl-3-methylammonium chloride, 0.1 mol/L Tris–HCl pH 8.0) was added and the reaction was stopped in a bath of boiling water for 5 min. The 3 mL detection solution (0.1 mol/L Tris–HCl (pH 8.0), 2 U choline oxidase, 2 U hydrogen peroxidase, 0.3 mg 4-amino antipyrine, and 1 mg phenol) was then added and shaken for 20 min at 200 r/min at 37 °C. The absorbance at 500 nm was measured and the enzyme activity was calculated using a standard curve of choline chloride as an assay mixture instead of the enzyme solution. One unit (U) of PLD was defined as the amount of enzyme that liberated 1 µmol of choline per min.

### Dry cell weight determination

After the fermentation process was completed. 30 ml of shaken fermentation broth was centrifuged at 6000×g for 15 min with a CF16RN refrigerated centrifuge (Himac, Japan) to collect the bacteria, washed twice with sterilized water (centrifugation conditions as above), and dried in an oven at 65 °C until constant weight. PLD production efficiency was defined as mg/g (dry cell weight).

### Optimization of carbon source and nitrogen source for SK-3

The fermentation medium was prepared using different carbon sources (sucrose, fructose, maltose, glucose, mannitol, soluble starch) and different nitrogen sources (beef extract, peptone, beef extract/peptone, ammonium sulfate, casamino acids, and ammonium sulfate/casamino acids). After 72 h of fermentation, the PLD enzyme activity and dry weight of the bacteria were determined. The best carbon and nitrogen sources were refined further. Mannitol, the optimum carbon source, was set at 7.5, 10, 12.5, and 15 g/L, and the ratio of ammonium sulfate to casamino acids (total 10 g/L) was set at 6:4, 5:5, 4:6, and 3:7 to study their effects on the expression of PLD in the recombinant strain SK-3.

### Optimization of incubation temperature and Mg^2+^ content

To further improve the production efficiency based on the above optimization, the effect of fermentation temperature at 28 °C, 30 °C, 32 °C, and 34 °C on the expression of PLD by the recombinant SK-3 strain was investigated. In addition, the concentrations of Mg^2+^ set at 0.3, 0.6, 0.9, 1.2, 1.5, 1.8, and 2.1 g/L were investigated for the effect of the PLD expression.

## Data Availability

All relevant data generated or analyzed during this study are included in this published article.
